# Mitochondrial Transfer in the Neurovascular Unit, Not Only for Energy Rescue: A Systematic Review

**DOI:** 10.14336/AD.2024.0461

**Published:** 2025-07-16

**Authors:** Daqiang Zhou, Sibo Yang, Jiehong Wu, Yanan Li, Huijuan Jin, Yan Luo, Feng Zhang, Junjie Jiang, Bo Hu, Yifan Zhou

**Affiliations:** Department of Neurology, Union Hospital, Tongji Medical College, Huazhong University of Science and Technology, Wuhan 430022, China.

**Keywords:** mitochondrial transfer, neurovascular units, tunneling nanotubes, microvesicles, mesenchymal stem cells

## Abstract

Despite substantial evidence highlighting molecular communication within the components of neurovascular units (NVU), the interactions at the organelle level have been insufficiently explored in recent decades. Mitochondria, for instance, beyond their traditional role as energy supply for intracellular metabolism and survival, provide a novel perspective on intercellular connections through mitochondrial transfer. These transferred mitochondria not only carry bioactive molecules but also signal to mitigate risks in both healthy and pathological conditions. In this review, we summarized mitochondrial transfer events, relevant routes, and underlying molecular mechanisms originating from diverse cell populations within NVU. We particularly focus on the therapeutic potential of this mechanism in treating central nervous system disorders, notably neurodegenerative diseases marked by mitochondrial dysfunction and then highlight the promising prospects of exogenous mitochondrial supplementation as a treatment target.

## 1. Introduction

Amongst the organs with the highest energy consumption, particularly the brain and spinal cord, there is a notable absence of energy storage compartments similar to adipose cells. Consequently, despite their diverse pathological characteristics, various neurodegenerative diseases share the common underlying pathogenic mechanism: disturbances in energy metabolism. The extensive energy consumption of nervous system highly depends on a sustained supply of oxygen and glucose from blood flow, which require a precise coordination between neurons and blood vessels [1, 2]. The emergence of the neurovascular unit (NVU) that is comprised of neurons, astrocytes, microglia, pericytes, and specialized endothelial cells, together with the extracellular matrix, has provided a framework for the targeted regulation of energy metabolism [3]. Disruptions in energy acquisition and utilization inevitably impair the central nervous system in terms of its homeostasis.

During various neurodegenerative diseases where energy metabolism disturbances occur, mitochondria typically bear the brunt. Hence, their abnormality has been identified as a conspicuous indicator of certain diseases. Mitochondrial dysfunction is mediated by various factors, including abnormalities in mitochondrial structure and dynamics [4], as well as the membrane potential [5], in addition to the opening of mitochondrial permeability transition pores (mPTP) [6]. Fortunately, cells initiate intercellular mitochondria transfer as a remedial measure. Over the past decades, organelle transposition between distinct cell types within the NVU has been observed in various conditions [7]. The significance of mitochondrial transfer involves the degradation of damaged organelles, energy rescue and cell survival, inflammation propagation and cancer proliferation. Given the tissue- and cell-specific molecular mechanisms of mitochondrial transfer, this review focuses on how various cellular components within the NVU perform mitochondrial transfer via tunneling nanotubes (TNTs), extracellular vesicles (EVs) or, sometimes gap junctions. However, endogenous mitochondrial transfer is constrained by the mitochondrial inventory of the neurovascular unit. The supplementation of exogenous mitochondria partly compensates for the deficiency of endogenous transfer. Therefore, we also highlight the therapeutic benefits of cell-independent mitochondrial transplantation and emphasize novel targeted interventions that combine mitochondrial transplantation with transfer.

## 2. Mitochondria and energy metabolism in the NVU

In adults, the brain represents a mere 2% of full- body weight, whereas it accounts for 25% of glucose and 20% of total oxygen consumption [2]. Glucose, as the primary source of energy, along with small amounts of alterative substrates, like ketone bodies or lactate, is converted to adenosine triphosphate (ATP), the universal biological currency, to support nerve conduction and non-signaling pathways. Specifically, ATP is primarily used to power sodium-potassium and calcium pumps for ionic gradients inside and outside cells [8]. Indeed, the metabolic profile among different cell types varies greatly from primary energy substrate to means of extracting ATP among different cell types, which may underlie the biological basis for mitochondrial transfer.

Compared to anaerobic glycolysis, mitochondria serve as highly efficient sites for extracting biological energy from glucose via the tricarboxylic acid cycle (TCA, also known as the citric acid cycle or Krebs cycle) and oxidative phosphorylation. As primary beneficiaries in an energy-demanding system, neurons drive glucose uptake from the blood, based on their energy needs rather than the blood glucose concentration [8, 9]. Glucose travels from capillaries to neurons either through direct diffusion in extracellular space or transshipment in astrocytes. Once glucose enters cells, it is predominantly broken down via oxidative phosphorylation (a process whose completion requires oxygen and proceeds within the inner mitochondrial membrane). Consistently, neurons equip with fewer phosphorylated pyruvate dehydrogenase (inactive form) and a faster TCA cycle to link up with glycolysis pathways in the cytoplasm [10]. Additionally, neural activity in energy-intensive synapses heavily relies on the rapid supply of ATP from aerobic glycolysis. Even glutamate released during nerve conduction, as stated in the astrocyte-neuron lactate shuttle hypothesis, can stimulate aerobic glycolysis in astrocytes, which subsequently generates lactate that is transported back to nearby neurons, and similar processes occur in oligodendrocytes [11]. In fact, energy demands of astrocytes are primarily supported by aerobic glycolysis [12]. Evidence suggests that mitochondria are present throughout astrocytes, from cell bodies to endfeet, with a mitochondrial volume/cell volume ratio comparable to excitatory neurons [13]. This indicates that mitochondria in astrocytes may remain a surplus station. Sometimes, as the main site of glycogen synthesis and storage in the NVU, astrocytes can increase glycolytic flux and respond to local energy demands without sufficient glucose supply or the activity of hexokinases [14]. Unlike versatile astrocytes, generally, microglia seldom accommodate neuronal energy requirements directly and also prefer oxidative phosphorylation pathways. However, when transformed into pro-inflammatory microglia due to neuroinflammation, their glucose metabolism undergoes a significant shift to enhanced anaerobic glycolysis [15]. Nevertheless, as the first element in the NVU to come into contact with blood, endothelial cells, with their predominantly anaerobic glycolysis-based energy metabolism, undoubtedly save more oxygen for other cell populations and meanwhile curtail reactive oxygen species (ROS) yield. These activities prevent potential cellular dysfunction [16]. Lactate derived from endothelial cells can be accepted by neighboring pericytes and employed for energy supply as well as amino acid biosynthesis. Interestingly, it is based on the fact that the glucose uptake of pericytes is four times that of endothelial cells. Furthermore, this energy substrate serves as a vital element in maintaining foolproof coverage of pericytes and moderate permeability of the BBB [17]. With energy constraints, for example, a long fasting, endothelial cells and astrocytes can take up ketone bodies (used in mitochondria) from the blood by monocarboxylate transporters, which provide more than 50% of gross energy in the brain. Thus, the presence of mitochondria caters to bioenergetic needs of various cell populations, even though they may not play a dominant role in some cell types.

Mitochondria, as highly dynamic organelles, constantly adjust their number, morphology, function, and distribution to meet the energy demands of cells [18, 19]. Processes such as mitochondrial fusion, fission, biogenesis, selective degradation, and transport support the preservation of mitochondrial stability and the regulation of chondriosomal quality [20]. Mitochondrial fusion involves the integration between inner and outer membranes of mitochondria, mediated by optic atrophy 1 (OPA1) and mitofusin 1/2 (MFN1/2) [21], respectively. A dual knockout of MFN1 and MFN2 precipitates a severe drop in oxygen consumption rates and levels of oxidative phosphorylation-related proteins [22], indicating a close relationship between mitochondrial dynamics and energy metabolism. Due to sharing metabolic substrates and mitochondrial matrix with each other, the deficiencies of partially damaged mitochondria are compensated for fusion with functional mitochondria, and this ultimately enables them to continue functioning within cells [23]. Conversely, fission removes unfunctional components including damaged proteins, impaired mitochondrial membranes, or abnormal mitochondrial DNA (mtDNA) [24]. The smaller functional mitochondria produced by fission are more mobile and are more likely to be concentrated in extremely energy-demanding intracellular regions [25]. Dynamin-related protein 1 (Drp1), in a guanosine triphosphate (GTP)-dependent manner, binds to receptors such as mitochondrial fission 1 protein (Fis1), mitochondrial fission factor (MFF), and mitochondrial dynamics proteins of 49/51 kDa (MiD49/51), thus initiating mitochondrial fission when recruited from the cytoplasm to the mitochondrial outer membrane [22, 25]. Drp1 is also recognized as a necessary factor that ensures brain development and synapse formation [26, 27]. Harmony between fusion and fission maintains the overall chondriosomal shape, preventing excessive elongation or fragmentation [28]. However, this stability may undergo adaptive changes when the bioenergetics in cells fluctuate [29]. Fusion fails to compensate for all mitochondrial defects, and fission cannot completely escape from the shadow of abnormal mitochondrial and organelle components. Mitochondrial biogenesis and selective degradation fulfill the basic requirement of maintaining continuous operation.

In response to increased energy demand or decreased levels in intracellular ATP, mitochondria undergo growth and division for amplification through a series of biological events, including the activation of nuclear transcription factors (eg, peroxisome proliferator-activated receptor-γ coactivator 1-α, PGC-1α), the synthesis and import of mitochondrial proteins, and the replication of mtDNA [30]. On the other hand, autophagosomes, double-membrane vesicles containing substrates, combine with lysosomes to effectively prevent the accumulation of useless mitochondria within cells [31, 32]. Autophagy, which is often observed following asymmetric mitochondrial fission, becomes activated because large, elongated mitochondria are not suitable for this process [33]. Additionally, MFN2, ubiquitinated by phosphatase and tensin homolog (PTEN)-induced kinase 1 (PINK1) and parkin, one kind of critical protein complex that mediates mitochondrial autophagy, inhibits mitochondrial fusion [34]. Therefore, the regulation of chondriosomal number, quality, morphology, and function is interdependent, and any disruption in these parts may cause energy metabolism disorders, at least to some extent.

## 3. Mitochondrial dysfunction in neurological and metabolic diseases

Flaws in mitochondria signal various cerebral diseases [35]. Disruptions in mitochondrial function accumulate over the prolonged lifespan of a cell or arises suddenly during metabolic disturbances. The extent of impairment can result in a range of outcomes, from mild, compensable energy metabolism anomalies to irreversible apoptosis. Calcium overload and mPTP opening, along with aberrant dynamics and excessive ROS production, converge to irreversibly damage mitochondria, driving disease progression.

The mitochondrial cascade hypothesis places mitochondrial dysfunction at a pivotal point in the progression of Alzheimer’s disease (AD), overlapping with other pathophysiological mechanisms [36]. Mitochondrial disorder is extremely marked in early AD where Aβ deposition and pathological tau protein exhibit a synergistic effect in this regard [37, 38]. In fact, Aβ accumulates within mitochondria before extracellular Aβ plaques form [39]. Initially, Aβ activates nicotinamide adenine dinucleotide phosphate (NADPH) oxidase 4 (NOX4) in pericytes, which is primarily localized to mitochondria [40, 41]. NOX4 mediates the generation of ROS and the release of endothelin-1 (ET-1). Then the latter acts on ET_A_ receptors in pericytes, leading to reduced capillary vasodilation, local cerebral blood flow, and energy supply [42]. In line with that, ROS generated by NOX eliminates nitric oxide (NO), a vasodilator, exacerbating neurovascular uncoupling [43]. It is Aβ-induced mitochondrial DNA damage in endothelial cells [44], accompanied by the release of endonuclease G and Smac [45] that worsens blood-brain barrier permeability. While mitochondrial fusion initially shows an upward trend transiently in astrocytes, due to an increase in the phosphorylation level of Drp-1 induced by Aβ, abnormal swelling and excessive fission ultimately appear. Even worse, phospho-Drp-1 transmits Aβ interference with mitochondrial function through extracellular vesicles and tunneling nanotubes from one neurovascular unit to another [46]. Moreover, Aβ can directly damage neuronal mitochondrial function [47]. The consequent oxidative stress in turn produces insoluble Aβ via upregulating the activity of γ-secretase [48]. That Aβ remaining inside chondriosomes interplays with cyclophilin D (CypD, a component of mPTP) causes mPTP to open. Still, a significant deficiency in CypD significantly improves mitochondrial stress together with capacity cognitive as well as synaptic in a mouse model of AD [49]. Interestingly, Aβ hyperpolarizes microglial mitochondrial membrane potential in a P2X purinoceptor 7 (P2X7R)-dependent manner, but does not result in expected high levels of ATP [50]. Mitochondrial autophagy disorder is another hallmark of AD [51]. Autophagic flux gradually decreases as substrate clearance becomes insufficient in lysosomes, such as microtubule-associated protein light chain 3II (LC3-II) and p62, although the formation of autophagic bodies and the genes associated with biogenesis are significantly upregulated in neurons from early AD patients [52]. Aβ can trigger an increase in intracellular calcium level [53]. On this basis, autophagosomes fail to fuse with lysosomes so that excessive dysfunctional mitochondria heap up in the cytoplasm [54]. A shortage in nicotinamide adenine dinucleotide (NAD), a necessity for glycolysis and mitochondrial respiration, also contributes to injured mitochondrial autophagy [54]. On the other hand, it has been revealed that the overexpression and phosphorylation of tau play a critical part in mitochondrial distribution and intracellular transport. Pathological tau initially promotes mitochondrial fusion to improve acute cellular apoptosis. However, in a later stage, abnormal interaction between it and Drp1 facilitates the fission course. The essential function of mitochondrial ATP synthesis is also severely restricted due to the inhibition of mitochondrial complex I and V, decreased activity in antioxidant enzymes, and depolarized membrane potential. Furthermore, mitochondrial dysfunction can lead to tau phosphorylation and aggregation [55]. This vicious cycle maintains neuronal tissue in a state of continuous damage, consistent with progressive Alzheimer’s disease.

Mitochondrial dysfunction, especially in the context of Parkinson’s disease (PD) where genetic factors are predominant, has been identified as a potential trigger for both motor and non-motor symptoms [56]. Mutations in nuclear and mitochondrial DNA affect various aspects including mitochondrial clearance, dynamics, energy production, and redox homeostasis [57]. Parkin, PINK1 and Parkinson disease protein 7 (DJ-1) from patients with autosomal recessive inherited PD build a direct relationship with mitochondria. Devitalized parkin, the most common autosomal recessive inherited PD-associated gene, is linked to the accumulation of toxic substrates parkin-interacting substrate (PARIS), for instance, an inhibitor of PGC-1α, which leads to mitochondrial biogenesis impairment [58]. Besides, a deficiency in PINK causes significant muscle weakness and dopaminergic neuronal degeneration. There also exists altered mitochondrial ultrastructure and functional impairment in drosophila model [59]. Although not as common, DJ-1 deficiency results in morphological mitochondrial alterations and affects respiratory chain complex integrity [60, 61]. Pathogenic variations in leucine-rich repeat kinase 2 (LRRK2), the most prevalent form of autosomal dominant inherited single-gene PD, impair both basal mitochondrial autophagy and PINK1/parkin-mediated mitochondrial autophagy initiation [62]. These mutants also disrupt the transport of mitochondria within neuronal processes [63]. Even in PD triggered by environmental factors, the disturbance of the respiratory chain complex remains a crucial characteristic [64]. Therefore, due to its metabolite in vivo 1-methyl-4-phenylpyridinium (MPP+), which leads to oxidative stress and calcium imbalance via targeting dopaminergic neurons in respiratory chain complex [65], 1-methyl-4-phenyl-1,2,3,6-tetrahydropyridine (MPTP) has become the gold standard for animal modeling in PD [66]. As PD progresses, mitochondrial dysfunction may worsen through the accumulation of α-synuclein in neurons. Abnormal α-synuclein aggregates tend to bind with mitochondria [67], and their interaction with the mitochondrial membrane can mediate mPTP opening and membrane potential reduction. Within the mitochondria, this can decrease the activity of electron transfer chain, followed by increased ROS and ultimately mtDNA damage [68]. Mitochondrial dysfunction also contributes to PD progression through mitochondrial damage-associated molecular patterns (DAMPs), such as mitochondrial transcription factor A (TFAM), cytochrome C, and cardiolipin, can be released into extracellular space where they initiate proinflammatory responses from glial cells and stimulate microglial phagocytosis, which finally result in disease progression [69].

Within minutes after cerebral ischemia, mitochondrial dysfunction occurs in the NVU. Calcium overload in the cytosol or mitochondria is an important factor that promotes mPTP opening [70]. Glutamate, due to the reduced activity of excitatory amino acid transporters 1/2 (EAAT1/2) on astrocytes, accumulates inside extracellular space [71, 72] and hyperactivates glutamate receptors on astrocytes in the penumbra, leading to a massive Ca^2+^ influx [73]. Mitochondria, whose function attach extraordinary significance to impressionable Ca^2+^ homeostasis, contain a highly selective mitochondrial calcium uniporter (MCU) that mediates Ca^2+^ uptake into the mitochondria [74]. In ischemic stroke, elevated chondriosomal Ca^2+^ levels result in the cleavage of BH3 interacting-domain death agonist (BID) into truncated BID (tBID), which interacts with B-cell lymphoma 2 (BCL2) antagonist/killer 1 (BAK), an apoptotic protein, at the mitochondrial membrane. Activated BCL2-associated agonist of cell death (BAD) relocates to outer mitochondrial membrane, inducing BCL2-associated X (BAX) to open mPTP [75]. In neurons under ischemic conditions, the accumulation of fatty acid-binding protein 3/5 (FABP3/5) triggers BAX oligomerization at the mitochondrial membrane, which also results in outer mitochondrial membrane permeabilization [76]. Moreover, it is reported that the toxic lipid peroxidation product 4-hydroxynonenal (4-HNE) that FABP3/5 mediates mainly damages mitochondrial membrane proteins [76]. Subsequently, the leakage of protons causes mitochondrial membrane potential dissipation and a narrower pH gradient, inhibiting the activity of the electron transport chain [77]. Evidence shows that a significant calcium ion influx induces an increase in the activity of calpains in neurons [78], especially calpain-1, which resides in the mitochondrial membrane space through its N-terminal mitochondrial targeting sequence [78]. Calpain-1 cleaves apoptosis-inducing factor (AIF) and facilitates its translocation from mitochondria to the nucleus [79]. Escaped cytochrome C and AIF cause DNA fragmentation and cell death via caspase-dependent and caspase-independent pathways, respectively [77]. In addition, succinate promotes rapid superoxide production from complex I after reperfusion, and the ensuing ROS in high concentration can sometimes contribute to continuous activation of mPTP and inner membrane anion channel, disrupting the mitochondrial redox state and further exacerbating ROS production. This ROS-induced ROS release theory makes mitochondria the main source of ROS during ischemic stroke [77]. ROS can activate matrix metalloproteinases that degrade extracellular matrix and increase BBB permeability, worsening the local microenvironment of ischemic region [80, 81]. Experimental results from both ex vivo and in vivo studies show that mitochondrial fusion is reduced, while fission activity increases, a disparity that is widened by reperfusion. [82]. The excessive fragmentation of mitochondria and the disappearance of mitochondrial cristae generate obvious abnormalities in mitochondrial structure, resulting in a sharp decline or even an interruption in oxidative phosphorylation coupling and ATP production. Worse still, dysfunctional sodium-potassium ATPase in turn exacerbates calcium overload, mPTP opening, ROS production, and changes in mitochondrial structure [20].

Mitochondrial energy metabolism disruption comes after two distinct nadirs during traumatic brain injury(TBI) [83]. Within just thirty minutes, the respiratory control ratio (RCR) in the impacted cortical region significantly decreases [83], indicating impaired coupling between oxidative phosphorylation and the electron transport system. Aberrant lipid metabolism in NVUs after TBI interferes with the function of the electron transport chain. The accumulation of ceramide within chondriosomes, triggered by the activation of neutral ceramidase (NCDase) [84] and acid sphingomyelinase (ASM) [85], causes a reduction in cytochrome c oxidase (complex IV) activity. Following a brief recovery, RCR undergoes a more pronounced second decrease, accompanied by a deficient capacity to buffer Ca^2+^ [83]. The ATP synthesis rate rapidly declines in response to the poor utilization of energy substrates in the damaged brain area, which is severely limited by dehydrogenase activity and coenzyme content in mitochondrial matrix or membrane, including NAD and flavin adenine dinucleotide (FAD), thereby inclining the overall NVU metabolic pattern toward anaerobic glycolysis [86]. At this stage, time-dependent mitochondrial microstructural damage becomes evident, with almost no normal-looking mitochondria observed in samples even twelve hours later [83]. Mitochondrial fragmentation is often associated with Drp1. Untargeted metabolomics analysis shows that 20-hydroxyeicosatetraenoic acid (20-HETE), a metabolite of arachidonic acid, is significantly elevated after TBI, leading to increased Drp-1 and ROS production by inhibiting sirtuin 1 (SIRT1)/PGC-1α and reducing mitochondrial membrane potential [87]. The overexpression of phosphoglycerate mutase 5 (PGAM5) in neurons plays a dysfunctional dynamic regulation role by assisting Drp1 phosphorylation and translocation to mitochondria [88]. Additionally, mitochondrial autophagy after TBI can exhibit either excessive activation or relative insufficiency, depending on the type and severity of injury [89, 90]. In a moderate TBI model, the inhibition of Drp1 results in lower levels of mitochondrial autophagy but exacerbates neuronal loss and behavioral defects in rats [91], indicating that despite a slight increase, mitochondrial autophagy remains relatively insufficient. Interestingly, according to another study, mitochondrial division inhibitor 1 (Mdivi-1) seems to have an opposite effect by inhibiting PINK1/parkin-mediated mitochondrial autophagy. Moreover, ASM is deemed to engage the impairment of mitochondrial autophagic flux [85]. In fact, a vital criterion of mitochondrial quality is mtDNA content. Elevated levels of cyclin D1 (CD1) following brain trauma suppress the acetylation of nuclear respiratory factor 1 (NRF1), reducing its transcriptional activity and leading to a decrease in mtDNA content [92]. The binding affinity between TFAM and the hypomethylated promoters (HSP1 and HSP2) in mtDNA during repetitive mild TBI is weakened, which further reduces the level of mitochondrial proteins encoded by mtDNA and mitochondrial biogenesis, resulting in ATP deficiency [93]. Furthermore, the activation of poly-ADP-ribose polymerase1 (PARP-1) following injury can interfere with mtDNA repair, exacerbating the situation [94].

Some vascular disorders not only directly precipitate dysfunction in mitochondrial activity within neurovascular units but may also act synergistically with CNS diseases to compromise bioenergetic metabolism. Mitochondrial dysfunction stands as a pivotal factor in the genesis of cerebral alterations associated with diabetes [95]. Anomalous fluctuations in blood glucose levels induce disruptions in cerebral mitochondrial function and redox equilibrium [96]. Perturbations in mitochondrial dynamics are a common feature within the brains of diabetic individuals. For example, T2DM-induced mitochondrial hyperfission and autophagy can instigate morphological alterations in mitochondria [97]. Impairment in insulin signaling, glucose metabolism, and deficiencies in cellular bioenergetics are posited as the underpinnings for the correlation between T2DM and neurodegenerative pathologies or age-related cognitive decline [95, 98, 99]. Insulin receptors exhibit widespread expression across various brain regions, particularly within cerebral microvessels [100]. In canonical insulin signal transduction, insulin engages with its receptors, prompting their phosphorylation, subsequent recruitment of insulin receptor substrate adaptor proteins, and activation of the PI3K/AKT pathway. PI3K/Akt activation facilitates glucose transporter proteins, augments neuronal glucose uptake, and prompts the binding of hexokinase II to outer mitochondrial membrane, thereby enhancing glycolysis [95]. Nevertheless, postmortem analyses of AD and PD patient brains evince diminished expression of insulin receptors [101]. And mitochondrial dysfunction correlates with accelerated cognitive decline in the aging diabetic brain. Some insulin sensitizer agents have significant positive effects on cognitive impairment, with their mediated improvement of brain mitochondrial function serving as an essential mechanism [102].

Chronic hypertension impairs cerebral autoregulation and neurovascular coupling, diminishing the brain's ability to respond to pathological conditions [103]. Studies with Positron Emission Tomography have demonstrated that hypertensive patients exhibit reduced cerebral glucose metabolism, particularly in brain regions susceptible to ischemic stroke, such as the watershed areas of the middle and anterior cerebral arteries. Further targeted difference analysis of mitochondrial respiratory complexes in spontaneously hypertensive rat models revealed assembly defects in mitochondrial respiratory complexes I, III, IV, and V [104]. Recent studies suggest that hypertension-induced mitochondrial dysfunction serves as a risk factor for central nervous system diseases, extending beyond cerebrovascular diseases. Cortical samples from hypertensive neurovascular disease rats showed that overexpressed Protein Kinase Cδ mediates excessive mitochondrial fission by phosphorylating Drp1 at the serine 579 site, leading to mitochondrial ultrastructural damage [105]. Conversely, a study by Carla L. Busceti and her team elucidated that upregulated levels of uncoupling protein-2 in the striatum of stroke-prone hypertensive rats effectively balanced mitochondrial dynamics and mitigated inflammation and oxidative damage by regulating OPA1 and FIS1 protein levels [106]. Additionally, hypertension has been shown to exacerbate the production of cytoplasmic and mitochondrial ROS in endothelial and smooth muscle cells following TBI [107]. Astrocytes, during depression with hypertension combined, promote neuroinflammation in response to mitochondrial damage, potentially due to mitophagy abnormalities mediated by suppressed Mfn2 [108].

Under physiological conditions, mitochondrial ROS within the neurovascular unit play a role in sensing lipid nutrients, and their production is related to changes in the intracellular redox state, with minimal cytotoxic effects [109]. However, when lipid abnormalities exceed individuals’ compensatory capacity, they can have devastating effects on mitochondria. In experimental rabbits subjected to a long-term cholesterol-rich diet, significant damage to the mitochondrial cristae structure and loss of matrix in the cerebral cortex were observed, along with extensive edema in the NVU [110]. Serum analysis from rats on a high-fat diet revealed a significant reduction in superoxide dismutase activity and glutathione peroxidase content, accompanied by a marked increase in malondialdehyde levels. This situation is particularly worse off when hyperlipidemia coexists with cerebral ischemia-reperfusion injury. In their cerebral cortex, lipid abnormalities significantly enhance the release of cytochrome c from mitochondria to the cytoplasm [111]. Moreover, hyperlipidemia can exacerbate cerebral infarct volume by disrupting mitochondrial dynamics and respiratory chain activity [112]. Epidemiological evidence indicates that hyperlipidemia is associated with an increased risk of Parkinson's disease. 27-Hydroxycholesterol (27-OHC), a cholesterol oxidation derivative, is elevated in the brains and cerebrospinal fluid of PD patients. Immunofluorescence studies have shown that 27-OHC-modified α-synuclein more readily colocalizes with neuronal mitochondria, severely interfering with mitochondrial fusion activities [113].

## 4. Mitochondrial transfer in the NVU

A stereotype that mitochondria are permanently bound to specific cells was overturned in 2006 when Spees et al. discovered that mitochondria migrated from mesenchymal stem cells to alveolar epithelial cells [114]. In the past decade, mitochondrial transcellular behaviors have gradually been recognized as universal biological events, especially among cell subpopulations in NVU, covering a wide spectrum of CNS diseases [7]. As mentioned above, the common denominator of neurological maladies, namely impaired energy metabolism, prompts widespread agreement on energy rescue strategies. But pharmacological approaches targeting single enzymes, receptors and proteins are gravely limited by their effectiveness and clinical application [8, 115]. In contrast, functional mitochondria themselves occupy a crucial position in the energy metabolism of the NVU, and their excellent dynamic properties allow for the suppression of damaged mitochondrial activities, impeding negative developments in different aspects. Moreover, clinical cases of mitochondrial transplantation in children with myocardial infarction have encouraged the consideration of mitochondrial transfer as an emerging tissue revitalization tool in neurological diseases [116, 7]. Spontaneous mitochondrial travel in the NVU plays a dual role: balancing the energetic risks in the microenvironment from multiple perspectives while also potentially contributing to deteriorative neuroinflammation [117] and tumorigenicity [118]. Therefore, increasing resolution into the pathways and mechanisms behind this mitochondrial phenomenon helps to avoid unexpected “surprises” and effectively rationalize interventions.

### 4.1. Neurons as the origin

Generally, healthy cells possess the capacity to degrade their mitochondria in the cytoplasm. However, at ONHs, dysfunctional mitochondria in retinal ganglion cells gather at axonal sites in contact with astrocytic processes, which then shape protrusions and separate from the axon to form a closed membranous detachment. They undergo degradation by fusion with lysosomes after internalization by astrocytes together with microtubules [119]. Serial block-face electron microscopy suggests that such a mechanism of mitochondrial degradation may also be present between 5-hydroxytryptaminergic neuronal axons and astrocytes in superficial layers of the cerebral cortex [119]. With approximately one million mitochondria being degraded at any given time at the ONHs, transmitophagy constitutes a major pathway for the disposal of mitochondria under physiological conditions [119]. Nevertheless, the purpose of delivering neuronal mitochondria does not always seem confined to the degradation of damaged mitochondria. In vitro experiments have shown that transferred mitochondria originating from neurons in normoxia exhibit negative results for the lysosomal marker lysosomal-associated membrane protein1 (LAMP1) in astrocytes and that these mitochondria are able to undergo the cell cycle and redistribute into daughter cells, although there are no significant cell cycle differences compared to astrocytes that do not contain transferred mitochondria [120]. Furthermore, the mitochondrial membrane potential from recipient cells even slightly increased, reflecting the possibility that healthy mitochondria from neurons may overall modify organelle function in astrocytes in a relatively non-emergency context.

Neuronal mitochondrial transfer appears to be more active during some neurological diseases. In the presence of AD, cell-cell mitochondrial exchange was significantly enhanced and showed a marked positive age-related dependence [121]. To foster a PD environment, dopaminergic neuronal degeneration was induced in mice using 6-OHDA, and broken axon terminals gradually evolved into globosomes over two to five days. This particular mediator, which is not present in physiological transmitophagy, aggregates mitochondria that have issued requests for mitochondrial autophagy, followed by generating immature autophagosomes, and initiates the first step in organelle metabolism. Unfortunately, spheroids do not provide all the tools needed for degradation, as astrocytic processes approach and invade spheroids, moving mitochondria within them to be degraded in soma, completing the course where cells clear away useless mitochondria without triggering neuroinflammation [122]. Furthermore, a neuron-astrocyte co-culture to simulate ischemic stroke in vitro demonstrates that neurons can release damaged mitochondria as a stress signal that is received by astrocytes. This is followed by a striking increase in astrocytic mRNA levels of mitochondrial Rho GTPase 1 (Miro1) and TFAM [123], symbols of mitochondrial generation in astrocytes. Especially within the first 24 hours after ischemia/reperfusion, the rate at which neurons release mitochondria into the extracellular space increases, correlating with the rapid accumulation of glutamate and ROS in the microenvironment. Therefore, not only does the movement of neuron-derived mitochondria imply the processing of obsolete organelles, but it also acts as an impressible receptor, communicating to other cell populations the current condition of the nervous system and prompting them to respond [123].

### 4.2. Microglia as the origin

Microglia, under normal circumstances, are capable of releasing functional mitochondria into extracellular spaces. However, those activated by neuroinflammation or neurodegenerative diseases release fragmented mitochondria instead of free mtDNA, which can propagate nerve injury independently [117]. Neurotoxins such as Aβ and polyglutamine induce microglial activation depending on immoderate mitochondrial fission mediated by Drp1/Fis1 [124]. Following activation, only 50% of mitochondria freed from microglia are functional, as indicated by the decreased mitochondrial membrane potential, reduced ATP levels, and increased ROS production [117, 125]. On the one hand, cell contacts between microglia and neurons allow bi-directional transfer of mitochondria and α-synuclein. Microglia transfer mitochondria preferably to α-Syn enriched neuronal cells over the healthy ones, but quantitative analysis shows that α-Syn aggregates seem to move predominantly from neuronal to microglial cells, likely as a potential rescue mechanism [126]. When primary astrocytes are cultured in media containing mitochondria released from microglia activated in a model of Huntington’s disease (HD) or lipopoly-saccharide (LPS)-induced neuroinflammation, their phenotype switches to the proinflammatory A1 type, with a several-fold increase in proinflammatory cytokines tumor necrosis factor alpha (TNF-α) and interleukin-1β (IL-1β), as well as exhibiting mitochondrial rupture, dysfunction, and exacerbated cell death [117]. Moreover, electron microscopy reveals that released mitochondria from treated astrocytes show outer membrane rupture and reduced cristae structure, and that these astrocyte-derived mitochondria can induce a five-fold increase in neuronal mitochondrial dysfunction and death [117]. The aforesaid fragmented mitochondria originated in activated M1 microglia can also be directly taken up by neurons. Unfortunately, their integration with indigenous mitochondria in neurons ultimately leads to the deterioration of ischemic injury progression, in concert with extreme morphological and structural abnormality of mitochondria and upregulation of apoptosis protein BAX [125]. It is worth noting that mitochondria-containing microvesicles (MVs) produced from traumatic brains induce microglia to polarize toward M1 type in a ROS-dependent manner, forming a vicious cycle that aggravates neuroinflammation and brain edema [127]. These detrimental mitochondria can even spread beyond the NVU, binding to platelets through phospholipid- platelet glycoprotein 4 (CD36) interaction and inducing their procoagulant activity, resulting in post-traumatic coagulopathy [128]. However, in a mouse model subjected to spinal cord injury, zinc promotes non-contact-dependent mitochondrial transfer from microglia to neurons via the SIRT3/Mfn2 pathway. This significantly improves neuronal energy levels and reduces oxidative stress [129].

### 4.3. Pericytes as the origin

In NVUs, pericytes, one of the primary endogenous donors of functional mitochondria for astrocytes, maintain the stability of the nervous system by reducing the latter’s apoptotic rate. In a three-dimensional BBB model that includes pericytes, astrocytes, and endothelial cells, confocal microscopy has shown healthy mitochondria actively moving from pericytes to astrocytes [130]. To amplify the potential effects, astrocytes were subjected to oxygen-glucose deprivation/reperfusion (OGD/R) to induce mitochondrial damage and then co-cultured with healthy pericytes. It was subsequently discovered that pericyte-derived mitochondria in astrocytes were significantly more abundant than in the control group. Due to the transfer of functional mitochondria, the apoptotic rate of astrocytes decreased dramatically. This rescue mechanism also exists in other cellular stress states, in addition to ischemic stroke. Similar effects have been observed in pericyte-derived mitochondria after astrocytes are treated with staurosporine, a stressor that affects mitochondrial function [130].

### 4.4. Endothelial cells as the origin

Endothelial cells (ECs) in the nervous system appear to be a preferential source of polarized mitochondria. In comparison to non-cerebral endothelial cells, brain endothelial cells have a cytoplasmic mitochondrial volume that is two to four times larger [131]. Transferred mitochondria can significantly affect the overall mitochondrial function in recipient cells. Microvesicles derived from human brain microvascular endothelial cells, containing polarized mitochondria and ATP synthase F1 subunit alpha (ATP5A), a catalytic subunit of ATP synthase, can significantly increase extracellular acidification rates and improve maximal glycolytic capacity in hypoxic brain endothelial cells. Meanwhile, basal and maximal oxygen consumption rates rise significantly, leading to a major restoration of mitochondrial respiration. These improvements have been shown to be dose-dependent on MVs [131]. However, a slight increase in proton leakage has been observed in recipient cells [131], indicating that mitochondrial function has not yet reached an optimal state and protons can still leak through mitochondrial membrane. Furthermore, when co-incubated with cortical and hippocampal slices from mice treated with ischemia, neurons are capable of taking up these microvesicles. When co-cultured with astrocytes in a basal state, endothelial cells also plays a serving role [130]. Sometimes, the stability of blood vessels requires mitochondria from neighboring cells for maintenance. The knockout of Rhot1 in osteocytes to inhibit mitochondrial transfer also leads to the regression of transcortical vessels [132].

### 4.5. Astrocytes as the origin

**Figure 1.**
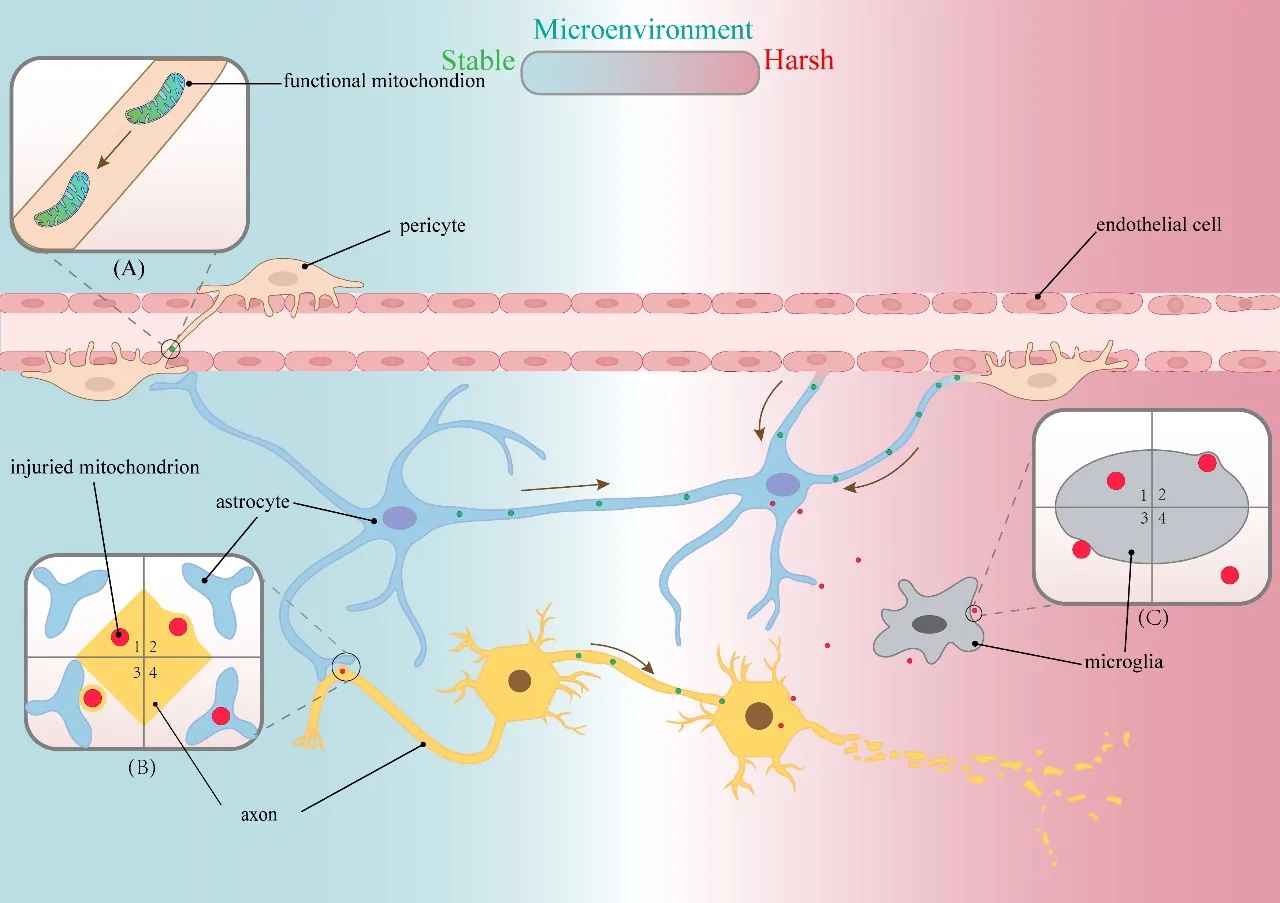
**An overview of mitochondrial transfer in the NVU**. Astrocytes serve as the center of mitochondrial delivery. (**A**) Mitochondria move in the TNT between pericytes, but do not enter the cell on the other side. (**B**) Astrocytes clear injured mitochondria from axons. (**C**) Microglia release dysfunctional mitochondria into the extracellular environment (for example, by exocytosis).

In the mitochondrial transfer network of NVUs, astrocytes act as a hub (Fig. 1), analogous to their anatomical location. They form unidirectional or bidirectional mitochondrial transfer chains with other cellular subpopulations, serving as a relay station for mitochondrial effects and allowing communication between distant cell populations. The dynamic migration of mitochondria among astrocytes makes them a powerful group with the ability to process mitochondrial distribution. Interestingly, this process can take place between human and mouse astrocytes, with a similar mitochondrial transfer efficiency to that between human and human astrocytes [120]. In various micro-environments, astrocytes spontaneously deliver functional mitochondria to neurons, suggesting that they have great potential as a temporary mitochondrial backup for neurons, especially in conditions of ischemic hypoxia. To track the destination of astrocytic mitochondria in vivo, photo-activatable mitochondria driven by AAV2-GfaABC1D-Cre was implemented. Subsequently, these labeled mitochondria were confirmed to appear in neurons and cerebrospinal fluid [133]. In mice cortical neurons subjected to OGR, a decrease in ATP levels and cell activity was reversed after extracellular mitochondrial particles derived from astrocytes were added to the culture medium, and protective effect disappeared when extracellular mitochondrial particles were removed [134]. However, it is worth noting that the control group intervened with liposomes containing ATP did not show a clear neuroprotective effect, demonstrating that the beneficial mechanism of mitochondrial transfer may not be restricted to the ATP supply [134]. The same team found that after injection of mitochondrial particles released from astrocytes into a mouse model subjected to middle cerebral artery occlusion (MCAO) for 24 hours, mitochondria entered neurons, accompanied by the upregulation of cell survival-related signals like phosphorylated protein kinase B (Akt) and B-cell lymphoma-extra-large (BCL-XL), and an increase in the mitochondrial marker translocase of outer mitochondrial membrane (TOM) [134].

Transcellular mitochondria can also play an antioxidant defense role in recipient cells. After cerebral hemorrhage, astrocyte-derived mitochondria enter neurons, restoring manganese superoxide dismutase (Mn-SOD) levels, preventing ROS-induced oxidative stress and neuronal death, and promoting the upregulation of synaptogenesis-related genes [135]. Astrocytic functional mitochondria have also been proven to have a positive influence on PD: in DA neurons exposed to rotenone, the mortality rate as well as the axonal length were strikingly reduced, but these neuroprotective effects were negligibly associated with nutritional or growth factors secreted by neighboring astrocytes [136]. In addition, mitochondrial damage in DA neurons promotes the efficiency of neuronal uptake of exogenous mitochondria [136]. Due to mitochondrial support from co-cultured astrocytes, neurons that undergo external force damage in TBI model partially allow their dendrite length to recover. Concurrently, levels in both mRNAs and proteins of mtTFA and PGC-1α, two critical activators of mitochondrial biogenesis, together with protein levels of TOM20 and mitochondrial cytochrome C oxidase II(mtCO2)involved in mitochondrial transport and energy supply, respectively, start to rebound [137]. Thus, astrocytes may promote neuronal transcription of mitochondrial synthesis-related genes in terms of mitochondrial biogenesis, while in mitochondrial transport, astrocytes focus more on translation or post-translational modification for relevant proteins.

Certain drug therapies, such as cisplatin and cobalt nanoparticles, induce neurotoxicity that can trigger intercellular communication at the organelle level. It is the disruption of neuronal mitochondrial function that contributes significantly to cognitive impairment following cisplatin chemotherapy. Abnormal neuronal intracellular calcium dynamics and the reduction in mitochondrial membrane potential move toward normalization after the transfer of mitochondria from astrocytes, and this is accompanied by an increase in neuronal survival [138]. Coincidentally, the inhibition of astrocyte-neuron mitochondrial transfer exacerbates mitochondrial morphological deformation, ROS production, ATP depletion, and even mitochondrial autophagy caused by cobalt nanoparticles [139]. A distinct difference in functional mitochondrial transfer from astrocytes to microglia compared to the aforesaid broken microglial resource also exists. Astrocytic transferred mitochondria carry humanin (HN), a small active peptide encoded by mitochondrial DNA, which, upon entry into microglia, upregulates its HN expression [140]. HN has been proven to help microglia switch to repairing phenotypes by promoting the expression of the transcription factor peroxisome proliferator-activated receptor gamma (PPARγ) and its target genes lipoprotein lipase and catalase. Microglial phagocytic activity is also positively affected by HN and transferred mitochondria, which in vivo manifests as accelerated hematoma clearance by clearing erythrocytes [140]. In fact, not all functional mitochondrial transfers are beneficial to the homeostasis of NVUs. In glioblastoma, the increase in tumor cell proliferation, migration ability, and resistance to chemotherapy drugs such as temozolomide, vincristine, or clomipramine may be related to astrocytic mitochondrial transfer [141]. To elucidate the mechanism by which the neurovascular unit enhances glioblastoma proliferation, Dionysios C. Watson and his team transplanted GFP-labeled syngeneic mouse GBM into the cranium of transgenic mice fluorescently labeled mitochondria. Stained tissue sections revealed that mitochondria from host cells entered the tumor cells through intercellular connections.15% -60% of glioblastoma cells receive mitochondria from non-malignant host cells, but sometimes before-mentioned mitochondria may shuttle through tumor microtubules to adjacent tumor cells after leaving the donor [118]. Subsequently, these cells achieved tumorigenic outcomes through more efficient mitosis and self-renewal abilities. And carcinogenic risks are believed to stem from overcompensation responses on hypoxia and metabolic remodeling caused by mitochondria [142].

Sometimes, transferred mitochondria may transcend the confines of the neurovascular unit and interact with other systems. When insulated by obesity, adipocytes experience oxidative stress, leading to the production of vesicles containing mitochondria. Due to decreased expression of CD36 and heparan sulfate proteoglycan on the surface, these vesicles could not be effectively phagocytosed and digested by resident macrophages. Consequently, these mitochondrial vesicles are released into the circulatory system. Microglia have been shown to phagocytose adipocyte-derived EVs [143]. Therefore, more research should be expanded on mitochondrial transfer between the neurovascular unit and other organs, despite the potential challenges. In the circulatory system, platelets can release MVs into the bloodstream under physiological conditions or certain specific situations. These MVs contain various platelet-derived RNAs, proteins, and mitochondria. These mitochondria have been shown to be involved in various cell-to-cell interactions, including vasculature, mesenchymal stem cells, and cancer cells. When exposed to PM2.5, a common harmful particle in the air, MVs released by platelets are absorbed by vascular endothelial cells. Subsequently, they induce endothelial apoptosis through classical cytochrome C/caspase-3 pathway [144].

## 5. Pathways and molecular mechanisms underlying mitochondrial transfer

### 5.1. Tunneling nanotubes

Tunneling nanotubes are dynamic tubular structures that extend from plasma membrane, rapidly forming within minutes and maintaining connectivity with distant cells for several minutes to hours [145]. Their F-actin-based skeletons, along with internal transport proteins work together to support the delivery of substances including organelles and biomolecules. Initially discovered in PC12 cells cultured in vitro in 2004, TNTs have since been extensively studied in various organs, such as the heart, lung, and kidney [146], to elucidate their pathological and physiological significance.

**Figure 2.**
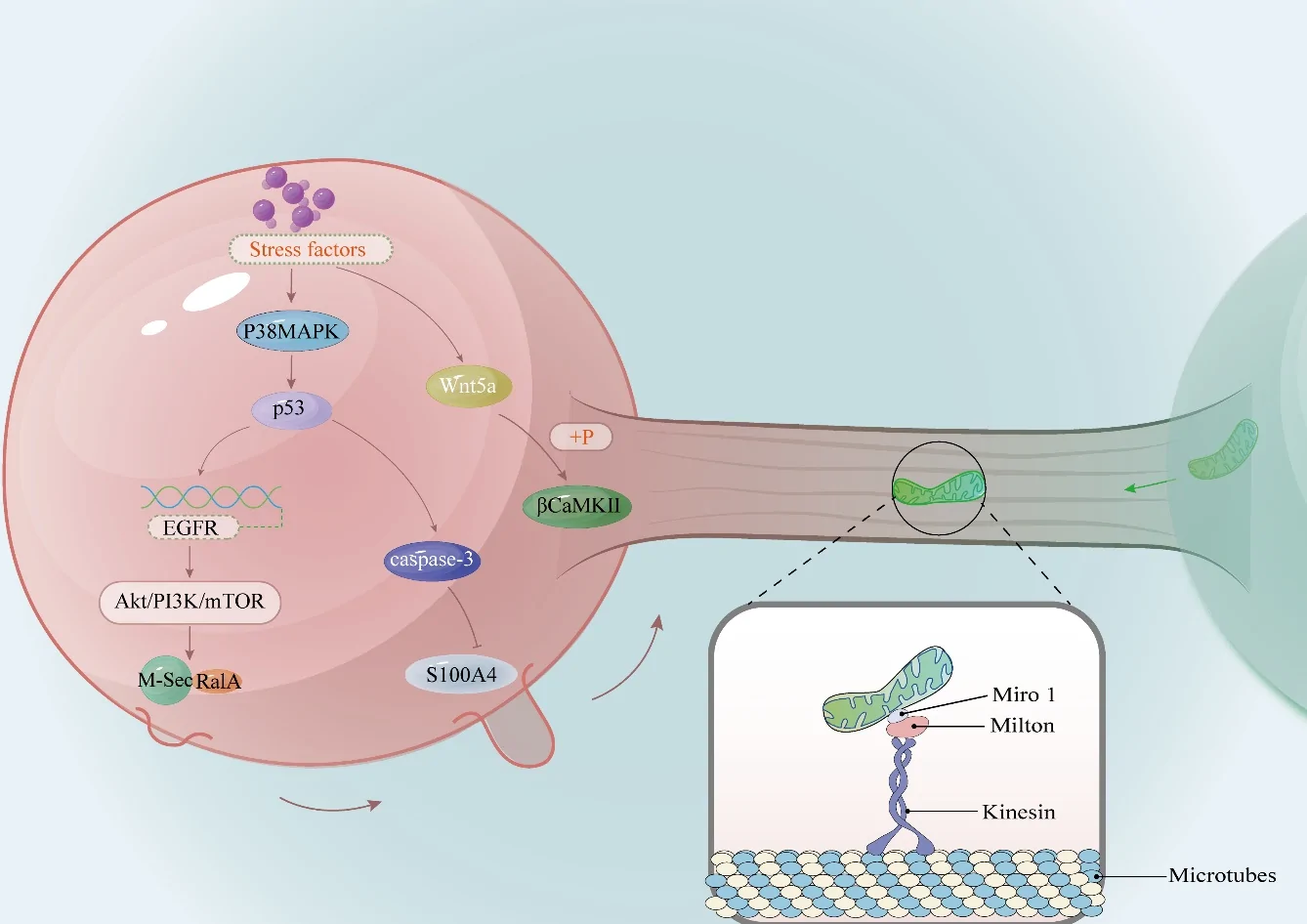
**The mechanism of mitochondrial transfer via TNTs**. After stresses such as ischemia, hypoxia and amyloid beta, the TNT initiates the process from the cell membrane via the Akt/PI3K/mTOR pathway. In the stressed cell, the difference in S100A4 concentration that is reduced by upregulated caspase-3 provides the TNT with a direction for targeting cells. During the extension of unfinished TNT, βCaMKII phosphorylated by Wnt5a separates from actins, which allows for the aggregation of actins and the formation of the skeleton. The partial enlarged view shows the detailed structure that helps mitochondria move in the TNT.

TNTs exhibit remarkable heterogeneity in their formation mechanisms across different cell types, which may contribute to their structural and functional diversity. The identity switch of various cellular components between donor and recipient cells within NVUs is accompanied by changes in the role of TNT formation and cargo transport rates. Therefore, it seems essential to focus on clues to how mitochondrial transfer occurs in NVUs (Fig. 2). Reportedly, factors that induce the formation of TNTs in astrocytes include H_2_O_2_, drugs, diseases, toxins, and misfolded protein aggregates. Among these, Aβ has a significantly stronger effect on inducing TNT formation in astrocytes compared to H_2_O_2_, STS, and glutamate [147]. Oxidative stress is a common inducer in TNT research. ROS or neurotoxic rotenone, for example, can activate p38 mitogen-activated protein kinase (p38 MAPK) in astrocytes and neurons, followed by higher activity in stress-related protein p53 [148, 149], a key regulator of TNT formation. On the one hand, p53 triggers tumor necrosis factor alpha-induced protein 2 (M-Sec) overexpression via the upregulation of epidermal growth factor receptor (EGFR) and subsequent phosphorylation of the Akt/phosphoinositide 3-kinase (PI3K)/mammalian target of rapamycin (mTOR) pathway [150]. In cooperation with RAS-like protein A (RalA) and the exocyst complex, M-Sec achieves actin polymerization on the cytomembrane and TNT formation [151, 152]. TNF-α can promote M-Sec expression through the activation of NF-κB, achieving similar effects [153]. On the other hand, caspase-3 activated by p53 in stressed cells promotes the cleavage of S100 calcium-binding protein A4 (S100A4), which builds a concentration gradient between stress (initiating) cells and target cells on the other side of the TNT, guiding TNT extension paths [147]. Furthermore, neuronal electrophysiological activities facilitate rapid TNT establishment with astrocytes by increasing the S100A4 concentration around neurons [147]. In 12-month-old 5×FAD transgenic mice (a common Alzheimer's disease model), S100A4 mRNA levels in astrocytes significantly increase, but no statistical differences are observed in 3-month-old and 6-month-old mice groups. Additionally, in this model, protein levels of Ambra1, a mitochondrial autophagy inducer, are doubled in astrocytes [121].

Although p53 plays a crucial role in the above process, multiple TNT formation mechanisms mediated by it are not even a supporting part in acute myeloid leukemia OCI-AML3 cells, human osteosarcoma cell line SAOS-2 cells, and pheochromocytoma PC12 cells [154]. Indeed, oxidative stress can induce TNT formation among PC12 cells, in a similar manner to astrocytes and primary neurons, with an active p38 MAPK/p53/caspase-3 pathway [155]. However, a study using ultraviolet light as an inducer suggests that some extra cysteine proteases are perhaps concerned with the establishment of microtubule-containing TNTs [156]. Furthermore, α-synuclein aggregates in PD often colocalize with transferred mitochondria within TNTs. These aggregates bind to outer mitochondrial membrane and then are transported together with mitochondria, contributing to disease spread. When donor astrocytes with LRRK2 mutations containing α-synuclein are co-cultured with wild-type recipient astrocytes, the efficiency of mitochondrial transfer significantly increases compared to recipient LRRK2 mutant astrocytes [157]. This indicates that astrocytic mitochondrial function and metabolic state are crucial prerequisites for their role as repositories for abnormal mitochondria. Moreover, the initiation of TNT involves the wingless-type MMTV integration site family, member 5A (Wnt5a)/calcium/calmodulin-dependent protein kinase II beta (βCaMKII) pathway. After ischemia-reperfusion, Wnt5a levels within neurons increase significantly [158], leading to the rapid but transient phosphorylation of βCaMKII. Phosphorylated βCaMKII detaches from actin, allowing for the entry of actin-regulatory proteins for actin polymerization. After a short time, dephosphorylated βCaMKII reattaches to actin, stabilizing the TNT skeleton and prolonging its half-life [159]. Additionally, myosin X (Myo10) in neurons participates in TNT formation in a non-Akt-dependent pathway. It is noteworthy that unlike the Wnt/βCaMKII pathway, Myo10 boosts the transfer of cargo from contributor to recipients [160]. This ability may be related to its cargo-binding capacity.

Mitochondrial transport via TNTs relies not only on actin cytoskeletons, but also on internal transport proteins. Miro1/2, Rho GTPases on the outer mitochondrial membrane, form complexes with molecular motors through the adapter protein trafficking kinesin protein1/2 (TRAK1/2) to enable mitochondrial movement along microtubules, a common event in axons. In astrocytes and neurons, levels of Miro1/2 in donor cells greatly affect intercellular mitochondrial transfer efficiency [138] while changes in levels of Miro1/2 in recipient cells seem to have no obvious effect on this process [120]. Following TBI, both mRNAs and proteins of Miro1 increase within neurons and astrocytes in the surrounding injured cortex [161], suggesting that mitochondrial transfer may be a self-rescue mechanism in TBI progression.

### 5.2. Extracellular vesicles

**Figure 3.**
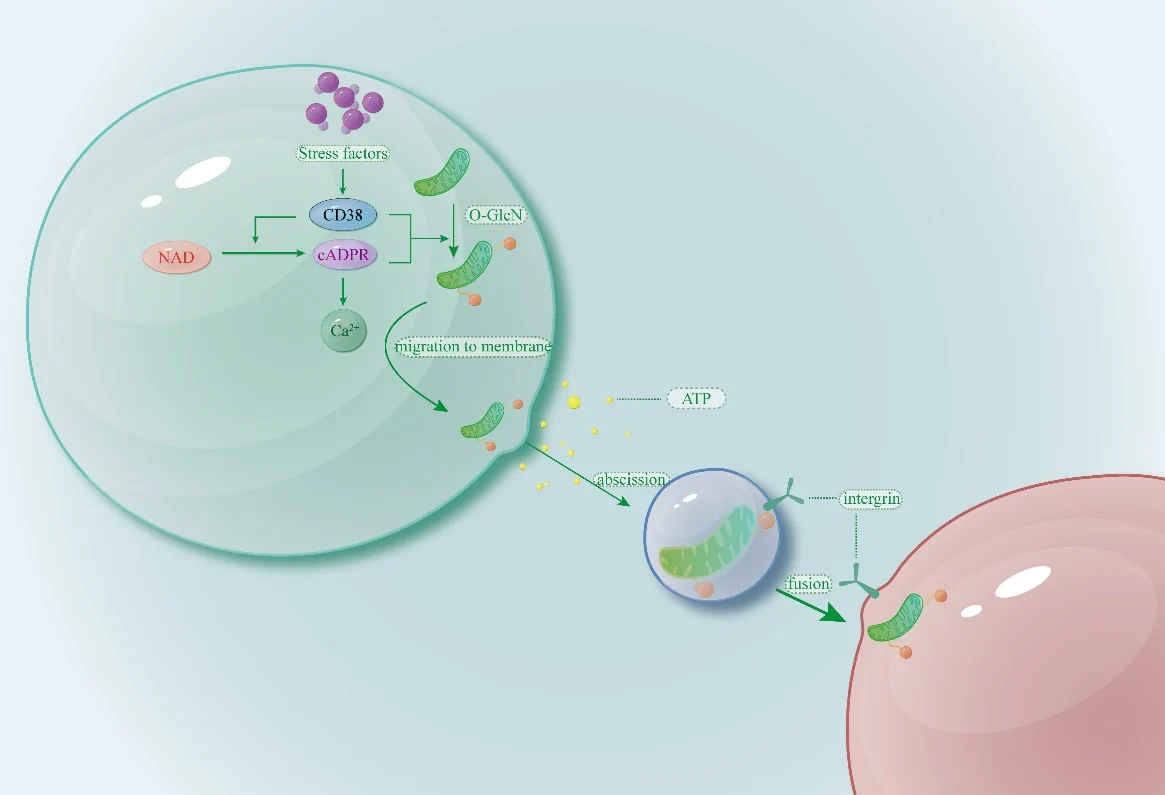
**The mechanism of mitochondrial transfer via MVs**. Increased transmembrane CD38 turns NAD into cADPR. Together they improve transferred mitochondrial quality by O-GlcNAcylation. Intracellular calcium ions that are elevated due to cADPR may attract the aforesaid mitochondria to the cell membrane. Excessive ATP freed from circumambient cells results in the abscission of vesicles containing mitochondria. Finally, these vesicles merge with target cells according to integrin-associated signals.

Extracellular vesicles can be divided into exosomes, microvesicles, and apoptotic bodies according to their biological functions, biogenesis, or size. It has been confirmed that all cell types in the NVU are capable of secreting extracellular vesicles. These nanoscale bilayer membrane structures mediate various biological events ranging from CNS maturation and development to synaptic signaling modulation, inflammation and cargo transport. As such, they are key participants in intercellular communication in the NVU. Sometimes, they can even penetrate the BBB into the bloodstream to influence peripheral tissues [162, 163]. Due to differences in the internal accommodation of diverse vesicles, their contents may vary from intact functional mitochondria to mitochondrial components such as mtDNA, NRF-1, ATP synthase ATP5A/ATPB and mitochondrial membrane protein voltage-dependent anion channel 1 [164, 165]. Hayakawa et al. have documented that mitochondrial particles released from astrocytes rescue neurons after a stroke[134]. Mitochondria derived from astrocytes are observed in large vesicles (Fig. 3), which also contain lipid droplets and ATP, with β1-integrin on their coat derived from cell membrane[166]. Following neural inflammation or aging, ADP-ribosyl cyclase 1 (CD38), a kind of transmembrane glycoprotein, is upregulated[167] and catalyzes the conversion of NAD to cyclic adenosine diphosphate ribose (cADPR) so as to increase the Ca^2+^ levels within astrocytes[134]. In models of diabetes-related cognitive dysfunction, the activation of phosphoserine phosphatase within astrocytes activates insulin signaling by dephosphorylating IRS1 at the Serine 789 site, thereby amplifying the expression of CD38[168]. The increase in Ca^2+^, together with the action of microtubules, leads to the translocation of mitochondria within astrocytes towards the cytomembrane[169], where they are captured and subsequently shed into the microenvironment by excessive ATP release or repetitive stimulation from neighboring reactive astrocytes[166].

When free large vesicles come into contact with neurons, they slide on neuronal surface through interactions between prion proteins (PrP) on the vesicle surface and PrP or other PrP-binding proteins to seek out preferred sites[170]. Additionally, Hayakawa et al. have suggested that the transfer of mitochondrial particles from astrocytes to neurons might involve an integrin-mediated non-receptor tyrosine kinase (Src)/spleen tyrosine kinase (Syk) mechanism[134]. During TBI, upregulated sigma-1R, which is located on the mitochondrial-associated membrane (MAM) and regulates communication between the endoplasmic reticulum and mitochondria through its chaperone activity[171], enhances CD38 expression by activated extracellular regulated protein kinases 1/2 (ERK1/2) due to interaction with sigma-1R[172]. This promotes the transfer of mitochondria from astrocytes to neurons, which has been shown to have an antidepressant effect[172]. On the other hand, the CD38/cADPR pathway also makes a significant contribution to the regulation of transferred mitochondrial quality. Under normal conditions, the O-linked-N-acetylgluco-saminylation (O-GlcNAcylation) level of mitochondrial proteins in astrocytes remains very low. By driving mitochondrial protein O-GlcNAc, this pathway improves membrane potential and mtDNA content in those transferred mitochondria, which are then more likely to remain within neurons while the level of O-GlcNAcylation has no influence on the number of released mitochondria[173]. Mutations in glial fibrillary acidic protein (GFAP) in astrocytes, as seen in Alexander disease, trigger a significant adverse impact on the efficiency of astrocytic mitochondrial transfer though they fail to alter the number or properties of intracellular mitochondria in astrocytes. Notably, defects in the mitochondrial transfer efficiency should be repaired after the decrease in CD38 levels is reversed[120]. It is intriguing to note that the CD38/cADPR pathway can influence the process by which receptor cells internalize mitochondria-contained vesicles[174]. Within receptor cells, cADPR induces the dissociation of FK-506 binding protein 12.6 from ryanodine receptors, leading to the release of calcium stored within the endoplasmic reticulum. Excessive cytoplasmic calcium, upon binding to calmodulin, triggers conformational changes in F-actin, facilitating cytoskeletal remodeling and membrane invagination, consequently initiating endocytosis. The heightened endocytosis for astrocyte-derived mitochondria in U87 glioblastoma cells serves as compelling evidence. However, recently, Jian Zhou et al. presented a distinctly different perspective: in astrocytes, low-density lipoprotein receptor-related protein-1(LRP-1) promotes mitochondrial transfer from astrocytes to neurons by intervening in metabolic reprogramming. This process does not involve the CD38/cADPR mechanism. After ischemia-reperfusion injury, downregulated LRP1 leads to a shift towards glycolysis in astrocytic metabolic patterns, during which the excessively accumulated lactate promotes the lactylation of intracellular proteins, particularly ADP-ribosylation factor 1 (ARF1). Protein-protein interaction analysis categorized the top gene ontology biological processes for proteins interacting with ARF1, revealing that the relevant proteins are mainly involved in vesicle budding, transport, and localization processes[133].

### 5.3. Gap junctions

Gap junctions and hemichannels are essential components of the neurovascular unit, able to convert the prerequisite for local stability into increased resistance to damage or impaired resistance to metabolic disturbance and oxidative stress[175]. Each HC contains six connexin (Cxs) subunits, with two adjacent HC units on neighboring cell membranes forming homotypic or heterotypic GJs. In the NVU, there is a high-level expression of Cx43 in astrocytes[176], cerebral endothelial cells[177], activated microglia[178] and pericytes[179] while neurons show a preference for Cx26 and Cx36 or even Cx40[180]. In this regard at least, it is evident that Cx43, encoded by the gap junction protein alpha 1 (GJA1) gene, seems to be one of the most vital elements in neurovascular linkage. The critical point, however, lies in the fact that pores of gap junctions only allow the passage of substances under 1kDa[181]. Even with the remarkable flexibility of mitochondria, direct transfer through gap junctions remains incredible. A specific type of TNTs, inter-pericyte tunneling nanotube, has proximal openings while with only GJs comprising Cx43 for communication at the closed distal end. Although mitochondrial movement has been detected in these TNTs, crossing between cells is not achieved due to the restriction of GJs[182]. This seems to suggest that the classical pathway mediated by GJs cannot manage mitochondrial transfer. However, in vitro experiments have demonstrated that the overexpression of GJA1-20k in astrocytes enhances mitochondrial delivery between astrocytes and neurons, with the downregulation of phosphorylated Cx43 (non-functional form) and an almost constant level of Cx43[183]. Furthermore, after specific inhibition for Cx43 hemichannels, neurons in TBI almost completely lose this mitochondrial source[183], which hints that GJs are essential for mitochondrial migration under certain conditions.

In astrocytes, the phosphorylation state of Cx43 affects its turnover within cells. Dephosphorylated Cx43 remains stable in GJs and maintains its distribution on the cell membrane at cell-cell contacts. [184]. For this, one hypothesis demonstrates that Cx43-mediated mitochondrial transfer may involve TNTs, despite the lack of a direct binding site between Cx43 and F-actin. Drebrin, an F-actin-binding protein, co-localizes with Cx43 on the cytomembrane of astrocytes, especially at the blood-brain barrier (BBB). [185]. In addition to interacting with microtubules, Cx43 indirectly binds to F-actin through a complex with drebrin to control cytoskeletal rearrangement, but the binding is likewise regulated by the phosphorylation of Cx43, meaning that phosphorylation can disrupt the interaction between them[185]. Therefore, the dephosphorylation of Cx43 may promote the process of TNT extension. Another phenomenon observed in ovarian cells involves the transfer of mitochondria through GJs formed by Cx43. This process, called connexosome or annular gap junction formation, involves one of the two connected cells engulfing the GJ and using the adjacent cell’s membrane and cytoplasm to form a double-membrane vesicle[186]. However, whether similar mechanisms exist in the NVU remains to be determined.

## 6. Treatment

### 6.1.1. Facilitating mitochondrial transfer in the NVU

Neural energy demands are met by a vast reservoir of mitochondria, which can be redistributed through mitochondrial transfer to maximize energy supply to diverse cell populations and prevent them from reaching a state of death or apoptosis (Table 1). Endogenous mitochondrial transfer proves itself as one of the primary ways to replenish mitochondria in stressed cells, while maintaining the integrity of mitochondrial function in donor cells is a necessary background for this process. A1 astrocytes, unlike typical astrocytes, release dysfunctional mitochondria that, upon entering neurons, integrate into the neuronal mitochondrial network and exacerbate mitochondrial damage. A heptapeptide cargo loaded within macrophage-derived exosomes has been shown to normalize the release of mitochondria mediated by the excessive interaction of Drp1/Fis1, reducing A1 astrocyte activation and effectively increasing the number of healthy mitochondria moving into neurons[187]. Ginsenoside Rb1, in a CD38-dependent manner, reversibly inhibits the activity of electron transport chain complex I followed by alleviated ROS production and calcium overload. At the same time, reactive astrocytes are curbed. These undoubtedly contribute to normalizing astrocytic mitochondrial membrane potential and preventing mitochondrial fragmentation, which provides neurons after stroke with more functional mitochondria[188].

An intervention with melatonin tightly links mitochondria among neurons. After melatonin administration, the number of TNTs between normal N2a cells and mtDNA-depleted ones rises sharply, and mitochondrial fusion occurs more frequently in the latter. When this effect is projected in vivo, the result shows a reduction in the area of brain infarction and neuronal mortality, as well as improvement in brain edema and myelin integrity[189]. Specifically, after reperfusion, melatonin completely reverses the downregulation of TOM20 and translocase of the inner membrane (TIM23, import receptor subunits) and the matrix protein HSP60, even with a higher level than control groups[189]. Due to levels of fusion and fission factors approaching their counterparts in control groups without OGD/R treatment, mitochondria treated with melatonin appear tubular and elongated[190]. That is to say, the morphology and dynamics of mitochondria are significantly improved. Additionally, physical treatment can sometimes boost the efficiency of mitochondrial transfer. Mild temperature reduction, 33°C, for example, can promote astrocytes to spontaneously release more functional mitochondria into the culture medium, which neurons then take up, leading to increased intracellular ATP levels, mitochondrial membrane potential, and cell viability[191]. Similarly, pre-treatment with hyperbaric oxygen therapy, results in more frequent mitochondrial transfer from astrocytes to neurons, increasing neuronal survival[192].

**Table 1:** Interventions facilitating mitochondrial transfer in NVUs.

Drugs/Interventions	Mechanisms	Results	Source of evidence	Ref.
**Heptapeptide**	Inhibit the interaction between Drp1 and Fis1	Produce less injured mitochondria in astrocytes More functional mitochondria move to neurons: less infarct size in brain	Cell co-culture in vitro/tMCAO model in rats	[187]
**Ginsenoside Rb1**	Activate CD38/cADPR/Ca^2+^ pathway	Suppress the activation of astrocytes and keep mitochondria in cells functional: Accelerate astrocyte-neuron mitochondrial transfer	Cell co-culture in vitro/cerebral ischemia model in mice	[188]
**Melatonin**	Upregulate the expression PGC1α and SIRT3	More mitochondrial transfers between neurons: More normal mitochondrial morphology: Less mitochondria-derived ROS	Cell-coculture in vitro/acute ischemic stroke model in rats	[189, 190]
**Hyperthermia (33°C)**	Facilitate astrocytes to release their mitochondria into extracellular space	More ATP and increased mitochondrial membrane potential in neurons: Decreased cell death rate	Cell-coculture in vitro	[191]

**Pretreatment with hyperbaric therapy**	Boost mitochondrial transfer from astrocytes to neurons	Increased neuronal viability	Cell-coculture in vitro	[192]

Abbreviations: Drp-1, dynamin-related protein 1: Fis1, mitochondrial fission 1 protein: tMCAO, transient middle cerebral artery occlusion: CD38, ADP-ribosyl cyclase 1: PGC1α, peroxisome proliferator-activated receptor-γ coactivator1-α: SIRT3, sirtuin 3: ROS, reactive oxygen species: ATP, adenosine triphosphate.

### 6.1.2. Clinical transformation challenges

When a research team discovers a candidate drug that can promote mitochondrial transfer, the first phase of drug development involves synthesis processes, toxicology, pharmacology, pharmacokinetics, and formulation development. Once the candidate drug passes preclinical trials, an "Investigational New Drug" application must be submitted to the regulatory authorities, such as the (C)FDA, to allow human testing. Upon approval, the drug undergoes Phase I, II, and III clinical trials.

In brief, Phase I clinical trials are preliminary pharmacological and human safety evaluations conducted on healthy volunteers to assess the drug's tolerability and pharmacokinetics. Phase II clinical trials typically involve randomized blind controlled trials to preliminarily evaluate the drug's efficacy and safety in patients. Phase III clinical trials involve larger groups of patient volunteers in multicenter trials to confirm therapeutic effects, providing crucial data for the drug's registration application and approval.

However, clinical translation faces numerous unforeseen challenges. The process from experimental results to clinical translation is usually lengthy, often taking years and requiring substantial financial investment, which is typically beyond the capacity of ordinary research teams and biotech companies. During clinical research, it may be discovered that the new drug performs poorly in humans or shows no significant difference compared to a placebo. Some side effects, not observed in cell and animal studies, may become prominent during clinical trials. These discrepancies are often attributed to the significant physiological differences between animals and humans.

### 6.2. Mitochondrial transplantation

All cell types of NVUs have exhibited the ability to take up mitochondria in in vitro experiments[193], leading to the emergence of the idea of directly supplementing exogenous mitochondria while weakening the concept of donor cells. However, it is noteworthy that the de-emphasis of donor cells only means no longer emphasizing the temporal and spatial connection between donor and recipient cells, rather than completely ignoring the source of mitochondria. Exogenous mitochondria may come from different individuals or even species and may be utilized in either a bare or wrapped form (Table 2). These differences may cause deviations in transfer efficiency and intervention effects, but cellular components of the NVU still occupy the key position in this challenge.

**Table 2:** Mitochondrial transplantation targeting CNS diseases.

Target cells	Mitochondrial sources	Applications	Effects	Evidence	Administration routes	Ref
**Neurons**	Homogeneous dental pulp stem cells	AD	Increased neurite length: Decreased ROS	Aβ/STZ-induced AD model in vitro	/	[195]
	Homogeneous brain	AD	Improved cognitive performance: Promote autophagy in recipient cells	Aβ-induced AD model	tail vein injection	[194]
	Hela cells	AD	Improved cognitive performance: Reduced loss of hippocampal neurons:	Aβ-induced AD model	Intravenous injection	[196]
	Homogeneous astrocytes	PD	Increased neuronal survival rate	Cell-coculture in vitro	/	[199]
	Homogenous liver	PD	Better rotational movement and motor behavior: Improved mitochondrial function: Reduced level of serum inflammatory cytokines	6-OHDA induced PD model	Intranasal administration	[197]
	PC12 cells/heterogeneous osteosarcoma hybrids	PD	Reduced loss of DA neurons: Improved rat spontaneous movement	6-OHDA induced PD model	Injection in medial forebrain bundle	[198]
	Homogeneous hepatoma cells	PD	Increased activity of ETC: better motor function	MPTP-induced PD model	Tail vein injection	[223]
	Homogeneous astrocytes	IS	Increased neuronal viability: Decreased infarct volume	Cell-coculture in vitro/MCAO model	Stereotactic injection in corpus striatum	[199]
	Heterogeneous renal fibroblasts	IS	Reduced infarct size: Better motor function	Cell-coculture in vitro/MCAO model	Stereotactic injection in corpus striatum/injection via femoral artery	[200]
	Heterogeneous astrocytes	Cerebral hemorrhage	Recover the level of Mn-SOD in neuronal mitochondria: Prevent oxidative stress induced by ROS and neuronal death: Upregulate genes related to axon growth and synaptogenesis	ICH model	Intravenous injection	[135]
	Homogeneous liver/autologous muscle	TBI	Decreased neuronal apoptosis: recover ATP level: Downregulate TOM20 and p-JNK	Cell-coculture/CCI model	Target-injection in cerebral cortex	[193]
	Homogeneous brain	TBI	Recover long-term depression	CCI model	Injection in cerebral cortex	[201]
	Homogeneous macrophage	Neurotic pain	Diminish inflammatory hyperalgesia	Transient inflammatory pain models	Intrathecal injection	[202]
**Astrocytes**	Autologous muscle	IS	Reduce the formation of glial scar: Promote neurogenesis	MCAO model	Injection in lateral ventricle	[208]
	Homogeneous liver/Autologous muscle	TBI	Regulate the expression of BDNF in astrocytes	CCI model	Target-injection in cerebral cortex	[193]
	Homogenous hippocampus	Major depressive disorder (MMD)	Reduced reactive astrocytes: Regulate BDNF	LPS-induced depression model	Intravenous injection	[204]
**Microglia**	Heterogeneous renal fibroblasts	IS	1-Boost microglial proliferation and activation	MCAO model	Stereotactic injection in corpus striatum/injection via femoral artery	[200]
	Homogeneous blood and cerebrospinal fluid	TBI	More microglia turn to phenotype M1: Regulate iNOS: More serious encephaledema	Cell co-culture in vitro/Mouse Model of Fluid Percussion Injury	Injection in caudal veins	[127]
	Homogeneous hippocampus	MMD	Inhibit microglial activation: Downregulate IL-1β/TNF-α/COX-2	LPS-induced depression model	Intravenous injection	[204]
**Endothelial cells**	Homogeneous brain	TBI	Promote neovascularization:upregulate TJPs: Alleviate encephaledema	Cell co-culture in vitro/CCI model	Injection in cerebral cortex	[201]

Abbreviations: AD, Alzheimer’s disease: ROS, reactive oxygen species: Aβ, amyloid beta: STZ, streptozocin: PD, Parkinson’s disease: 6-OHDA, 6-hydroxydopamine hydrochloride: ETC, electron transport chain: MPTP, 1-methyl-4-phenyl-1,2,3,6-tetrahydropyridine: IS, ischemic stroke: MCAO, middle cerebral artery occlusion: Mn-SOD, manganese superoxide dismutase: ICH, intracranial hemorrhage: TBI, traumatic brain injury: ATP, adenosine triphosphate: TOM20, translocase of outer mitochondrial membrane 20: p-JNK, phosphorylated c-Jun N-terminal kinase: CCI, controlled cortical impact: BDNF, brain-derived neurotrophic factor: LPS, lipopolysaccharide: MMD, major depressive disorder: iNOS, inducible nitric oxide synthase: IL-1β, interleukin-1 beta: TNF-α, tumor necrosis factor alpha: COX-2, cyclooxygenase-2: TJPs, tight junction proteins.

### 6.2.1. Target for neurons

Undoubtedly, the susceptibility of neurons renders them a prime target for interventions. Different AD models have showed a high level of convergence on benefits of mitochondrial transplantation for neuronal survival[194-196]. Isolated mitochondria trigger autophagy in neurons to clean Aβ protein and injured mitochondria via NAD-dependent SIRT1 signal[194]. In order to overcome BBB, intranasal administration and injection into medial forebrain bundle are applied in some PD models[197, 198]. As compensation for dysfunctional mitochondria, the loss of DA neurons began to get eased and rats behaved more like normal individuals. Astrocytes rescue neurons efficiently through endogenous mitochondrial shift has declared their tremendous potential as a source of exogenous mitochondria. For instance, homologous astrocytic mitochondria reinforce neuronal vitality and reduce infarct volume in ischemic stroke[199]. Surprisingly, normal neurons seem not to enjoy the relevant nutritional benefit. Besides, xenogenic mitochondria supplementation also facilitates the recovery of muscle strength in rats after one week, but the intracerebral injection route appears to be slightly superior to the arterial administration in terms of these beneficial effects[200]. And the above authors emphasize the necessity of normal electron transport chain and ATP synthesis in transplanted mitochondria for neuronal protection. In a cerebral hemorrhage model, astroglial mitochondria administered through the intravenous route prevent ROS-induced oxidative stress and neuronal death via restoring Mn-SOD in neuronal mitochondria, along with the upregulation of genes related to axonal growth and synaptic formation[135]. Even exogenous mitochondria supplementation from sources outside the nervous system also yields stunning outcomes. The fact that mitochondria derived from homologous liver and autologous muscle tissues enable ATP levels in injured cortex to reach an almost normal standard only three days after TBI, but with a significant reduction in TOM20 and phosphorylated c-Jun N-terminal kinase (P-JNK) levels, reflects the remission of mitochondrial dysfunction and ROS generation respectively[193]. In the hippocampus of a hemisphere that has suffered TBI, long-time depression associated with synaptic plasticity regarding learning and memory processes also shows improved results after exogenous mitochondrial supplementation[201]. In addition, mitochondria-containing vesicles secreted by macrophages solve a thorny problem, namely the pain after neuroinflammation. Depending on the interplay between OX-2 membrane glycoprotein (CD200) and CD200 receptor (CD200R), those transplanted mitochondria quickly but transiently suppress inflammatory hyperalgesia, although this specificity requires intact mitochondria because vesicles disrupted by ultrasound cannot affect pain perception[202].

### 6.2.2. Target for microglia

When the interaction between transplanted mitochondria and microglia represents their regulatory role in neuroinflammation, the microglial response to transplanted mitochondria also partly reflects their immunogenicity. In a brain subjected to MCAO, heterogenous mitochondria promoted significant microglial proliferation one week after transplantation, but this effect disappeared four weeks later[200]. Maintaining the efficacy of mitochondrial transplantation may therefore require multiple interventions. Following TBI, there are numerous morphologically normal extracellular mitochondria in cerebrospinal fluid and blood, which unfortunately activate BV2 cells via an ROS-dependent pathway and induce M1 pro-inflammatory phenotype transformation, ultimately exacerbating brain edema[203]. Consistent with the aforementioned aberrant microglial activation, the expression of inflammatory factor inducible nitric oxide synthase (iNOS) is upregulated, as revealed by RT-qPCR analysis[203]. However, in the LPS-induced depression model, the implantation of healthy mitochondria from the same species inhibits microglial activation and reduces the levels of inflammatory factors such as IL-1β, TNF-α, and cyclooxygenase-2 (COX-2)[204]. Therefore, the perfect functionality of transplanted mitochondria remains a precondition for treatment efficacy, and the source of the mitochondria also deserves further study.

### 6.2.3. Target for ECs

The impairment of mitochondrial function in cerebral endothelial cells is closely associated with the disruption of the BBB, whereas the repairment of the BBB helps maintain local homeostasis. Damage in the BBB and brain edema are pivotal markers of TBI prognosis. However, after mitochondrial transplantation, both brain fluid leakage and water content begin to decrease. Immunofluorescence staining for endothelial cell marker platelet/endothelial cell adhesion molecule-1 (CD31) shows a significant increase in neovascularization in the damaged area, while the expression of tight junction proteins in cerebral endothelial cells, including zonula occludens-1 (ZO-1) and claudin-5, is upregulated[201]. In this regard, the BBB has been reconstructed to a certain degree.

### 6.2.4. Target for astrocytes

It is the normality in astrocytic mitochondria that support astrocytes to regulate the microenvironment in the NVU and act as a mitochondrial transfer hub. In the brain, brain-derived neurotrophic factor (BDNF) is mainly expressed in astrocytes. Successful mitochondrial transplantation triggers a higher level of astrocytic BDNF in the controlled cortical impact (CCI) model, accompanied by the relief of trauma-induced anxiety, spatial memory, and cognitive function in mice[193]. Recent reports suggest that combination of BDNF and truncated tyrosine kinase receptor B (TrkB.T1) in astrocytes regulates neuronal activity and participates in energy expenditure as well as glucose homeostasis, ensuring that neuronal running matches mammalian metabolic demands[205]. There usually exist a regional declined BDNF expression in major depressive disorder (MDD), which contributes greatly to neuronal atrophy and inhibited neurogenesis[206]. Fortunately, deficiencies in BDNF in the hippocampus of the MDD model achieve recovery a day after injection of mitochondria. The number of reactive astrocytes that actively participate in CNS inflammation by expressing pattern recognition receptors and cytokine receptors[207] then successfully falls steeply[204]. What’s more, mitochondrial transplantation shows similar effects to conventional antidepressants in terms of pleasure loss and associated anxiety[204]. After ischemic stroke, glial scars induced by undue proliferation of reactive astrocytes lead to an enlargement of the infarct area and inhibition of axonal growth[80]. When mitochondria extracted from autologous pectoralis major enter the recipient through the lateral ventricle, both a dramatic decrease in GFAP-positive astrocytes in the penumbra and a corresponding increase in the migration of neural progenitor cells take place[208]. These signify brisk neurogenesis.

### 6.2.5. Attentions and challenges

Transitioning from laboratory research to clinical applications, mitochondrial transplantation as an alternative therapeutic approach has accumulated exciting efficacy. Due to semi-autonomous feature of transplanted mitochondria, they can integrate into the host, proliferate, and dynamically respond to external environments. Consequently, this robust adaptability endows mitochondrial transplantation with the potential to provide personalized solutions for patients. Dissatisfied with the limited clinical applications reported for myocardial infarction and reproductive-related disorders, numerous biotech companies are contributing to the expansion of mitochondrial transplantation therapy[209]. Despite promising intervention effects demonstrated in animal experiments concerning CNS diseases, several considerations need to be addressed before patient utilization. Further research should focus on gaining deeper insights into aspects such as the isolation, purification, and long-term preservation of mitochondria, appropriate routes and doses of administration, strategies for crossing the BBB and targeting lesions, as well as ensuring biocompatibility. In fact, patents have already been filed for mitochondrial isolation products even before fully revealing their therapeutic potential. Over the past decades, solutions for storing mitochondria have transitioned from physiological saline and cell culture media to respiratory buffer solutions containing various inorganic salts and sucrose. Some researchers have attempted to preserve isolated mitochondria under -80°C conditions. However, freeze-thaw mitochondria exhibited significant decreases in ATP content and membrane potential, failing to improve cerebral microcirculation and neurological function scores after cardiac arrest[210]. To enhance therapeutic efficacy, some cell or animal experiments have utilized high-dose culture medium supplementation or even in situ injections into lesions. However, clinical translation necessitates consideration of patient compliance. Recently, Ziyu Wu and colleagues successfully salvaged damaged cardiac tissue by orally administering enteric-coated capsules containing mitochondria modified with nanomotors[211]. Due to variations in mitochondrial sources, biocompatibility remains a significant concern. When transplanted mitochondria enter normal cells or non-target organs, they have not been reported to exhibit significant cytotoxicity. Injecting autologous mitochondria into rabbit myocardium in an ischemia/reperfusion model improved inflammation, and notably, no anti-mitochondria antibodies were detected post-transplantation[212]. Another study indicated that serum cytokine levels did not significantly change within one month after transplantation therapy, suggesting that exogenous mitochondria entering the host do not elicit a robust immune response to a certain extent[213]. Current clinical evidence primarily focuses on the use of autologous mitochondria, typically isolated from unaffected muscle tissue. Numerous preclinical studies reference mitochondria derived from liver organs or specific cell types, even including tumors. However, the optimal source for transplantation may need to be carefully considered based on the patient's or volunteer's physical condition and disease background. For instance, individuals with mitochondrial genetic disorders have mitochondria that are unsuitable for autologous transplantation and cannot serve as sources for other patients. For certain cancer patients, the introduction of exogenous mitochondria poses the risk of promoting tumor proliferation and metastasis. Considering ethical concerns and donor willingness, tissues such as skin, muscle, or oral mucosa may be more accessible mitochondrial sources in the future. Researchers should also invest more effort into understanding how donor factors such as sex and age impact transplantation efficacy. The detrimental effects of aging on mitochondrial function are well-documented, suggesting that elderly individuals should generally be excluded from donor lists. Additionally, individuals with systemic diseases such as diabetes, hypertension, and dyslipidemia are unsuitable as donors due to their mitochondrial abnormalities. Future researchers should explore whether mitochondria can serve as vectors for certain infectious diseases, such as HIV or hepatitis B virus. Until such risks are thoroughly investigated, clinicians should exercise caution when considering mitochondria from individuals with infectious diseases for transplantation.

## 7. Discussion

The transfer of functional chondriosomes may alleviate energy disorders, accelerate inflammation dissipation, and repair tissue damage. Unfortunately, most observations of these phenomena have been only in in vitro co-culture systems, and it remains to be confirmed whether mitochondrial cross-linking occurs between certain cell populations in the NVU in vivo under specific circumstances, and whether the magnitude of transfer varies. As things stand, although each cell subpopulation in the NVU may act as a functional mitochondrial donor, their propensity to give varies. Pericytes and endothelial cells tend to partake of their organelle while neurons and microglia are more likely to enjoy others’ contributions. And astrocytes are perfectly suited as hubs of the mitochondrial transfer network. The process of managing mitochondrial transfer involves several pathways, with TNTs and vesicles being primary conduits. Many similarities exist between the mechanism involving TNTs and mitochondrial transport within neuronal axons, suggesting that some molecular signals might be cross-reference. In addition, previous clues suggest that fluctuations in intracellular calcium ion concentration are relevant to vesicle-mediated mitochondrial transfer, and thus the role of other signals related to Ca^2+^ homeostasis in initiating transfer should have been expanded. Notably, pathological proteins such as α-synuclein, Aβ[214, 215] and prions[216] as well as viruses including herpesvirus[217] and HIV-1[218], can diffuse via TNTs or extracellular vesicles. These raise concerns about whether facilitating mitochondrial transfer might exacerbate the spread of intercellular injurants.

Mitochondria released into the extracellular space may also act as indicators. Mitochondria from the neurovascular unit likewise lingers in the cerebrospinal fluid and blood. In subarachnoid hemorrhage, astrocytic mitochondria are enriched in the cerebrospinal fluid, and their reduced membrane potential is associated with worse neurological deficits[219]. Similarly, in one study, mitochondrial membrane potential in the cerebrospinal fluid of patients appeared to have a negative correlation with clinical outcomes at three months and provided a profile of brain integrity and recovery status[220]. The measurement of activities of mitochondrial complex IV and V in neuron-derived extracellular vesicles in the blood successfully predicted brain and retinal atrophy status in multiple sclerosis[221]. Levels of electron transport chain and active SOD1 in these vesicles are also thought to reflect mitochondrial dysfunction in AD[222]. The extension of the indicative role of extracellular mitochondria to other CNS diseases may offer potential as an early or specific predictor in the future.

Mitochondrial transplantation may serve as a complementary means of mitochondrial transfer. The integrity and functionality of mitochondria are prerequisites to ensuring the neuroprotective benefits of mitochondrial transplantation. Extraneous mitochondrial particles do not actively cluster in a specific cell type alone, but are widely distributed among cells in the NVU. While all cellular components of the NVU can uptake mitochondria, their capabilities vary both in vivo and ex vivo. [193, 200]. Neurons exhibit a greater proportion of exogenous accumulation, likely due to their high metabolic demand and the spontaneous direction of mitochondrial transfer. Microglia, on the other hand, present a distinct performance in vitro and in vivo. With much higher gain in vitro than in vivo, it seems that exploring hidden mechanisms may help functional mitochondria assemble more efficiently in target cells. Given the low intracellular efficiency for exposed mitochondria[200], as well as the fact that normal cells demonstrate little neurotrophic benefit after receiving healthy mitochondria, meaningless affinity for non-target cells is undoubtedly a waste of mitochondrial supply.

Therefore, combining mitochondrial transplantation with internal transfer in neurovascular units could become a novel approach to utilize mitochondria to remediate the nervous system in the future. Facilitating the transfer within NVUs effectively compensates for the shortcomings of mitochondrial transplantation in reaching or enriching target sites, while exogenous padding for network capacity removes the limitation for local stocks, especially when there is an urgent need for mitochondria in a short period during the acute phase.
